# Integrating Genetic Diversity and Agronomic Innovations for Climate-Resilient Maize Systems

**DOI:** 10.3390/plants14101552

**Published:** 2025-05-21

**Authors:** Xin Li, Yunlong Li, Yan Sun, Sinan Li, Quan Cai, Shujun Li, Minghao Sun, Tao Yu, Xianglong Meng, Jianguo Zhang

**Affiliations:** Maize Research Institute of Heilongjiang Academy of Agricultural Sciences, Harbin 150086, China; maize_lee@163.com (X.L.); 13945699869@163.com (Y.L.); sunyan19850301@163.com (Y.S.); lee18686774002@126.com (S.L.); cq6539@163.com (Q.C.); lshj_750425@163.com (S.L.); sunminghao_yg@yeah.net (M.S.); yutaoweiwei@163.com (T.Y.); 18045141806@163.com (X.M.)

**Keywords:** maize, climate resilience, climatic change, genomic selection, multiomics, speed breeding, gene editing

## Abstract

Maize is a vital staple crop significantly affected by climate change, necessitating urgent efforts to enhance its resilience. This review analyzes advanced methodologies for maize improvement, focusing on the identification of genetic determinants through QTL mapping, candidate gene mining, and GWAS. We highlight the transformative potential of CRISPR gene editing for identifying key regulators in maize development and the utility of virus-induced gene silencing (VIGS) for functional genomics. Additionally, we discuss breeding strategies leveraging the genetic diversity of maize wild relatives and innovations such as speed breeding and genomic selection (GS), which accelerate breeding cycles. Marker-assisted selection (MAS) plays a critical role in developing superior maize varieties. The review also encompasses agronomic practices and technological innovations, including GS, aimed at climate mitigation. High-throughput phenotyping and omics-based approaches, including transcriptomics and metabolomics, are essential tools for developing climate-resilient maize. Climate changes have a significant impact on maize production and pose unprecedented challenges to its cultivation.

## 1. Introduction

Maize, scientifically known as *Zea mays*, is a basic crop in global agriculture that has provided sustenance to millions of people worldwide [1]. Maize’s origin was traced back to thousands of years ago in Mesoamerica. Since then, it has adapted significantly to diverse climates and soils. Today, maize is a fundamental crop in agricultural systems worldwide [2]. Other than being a primary food source, it is also a significant component used in various industries, such as serving as essential animal feed, and is increasingly utilized in biofuel production, indicating its economic and food security importance [3].

However, climate changes have a significant impact on maize production and pose growing challenges to its cultivation [4,5,6,7]. Climate change includes factors such as rising temperatures, erratic precipitation patterns, and soil degradation, which can destabilize global maize yields, exacerbate food insecurity, and jeopardize livelihoods [6,8]. Therefore, developing maize cultivars that can withstand these environmental stresses is urgent, necessitating a concerted effort to utilize inherent genetic diversity within maize germplasm [9,10]. Various methods have been employed to mitigate climate stress on maize. Traditional breeding methods select maize lines with increased tolerance to heat, drought, and soil salinity [11]. Marker-assisted selection (MAS) of breeding and genomics expedites this process by identifying stress-tolerant genomic regions [12]. Furthermore, high-throughput phenotyping can rapidly screen maize germplasm for stress-related traits [13]. Hybrid breeding programs utilize heterosis to develop hybrids with increased stress tolerance [14]. Moreover, agronomic practices like conservation tillage and precision agriculture have also enhanced soil health and resource use efficiency. In addition, biological interventions, such as inoculation with beneficial microbes, bolster maize resilience [15,16]. Farmers can make informed management decisions using climate-informed decision support systems. Using these approaches, stakeholders can establish a more climate-resilient maize production system. Recently, many studies have been focused on utilizing the rich genetic diversity of maize populations to breed cultivars with increased resilience against abiotic stresses. Climatic changes have exacerbated drought, heat, and soil degradation, thereby posing formidable challenges to traditional maize cultivation practices. However, multiple studies have highlighted promising avenues for employing the vast reservoir of genetic variability of maize germplasm to enhance tolerance against these environmental adversities. Since maize provides global food security, this adaptation is urgently required. Maize is a staple food for millions. Particularly in regions where alternative food sources are scarce, it ensures the nutritional well-being of the population. Moreover, maize cultivation forms the backbone of many agricultural economies, supporting livelihoods and fostering rural development. Therefore, the vulnerability of maize to climatic fluctuations can be observed across socioeconomic strata, indicating the critical need for proactive adaptation measures. By combining traditional breeding approaches with cutting-edge genomic technologies, we can enhance maize production. Innovative agronomic practices and climate-informed decision support systems are also vital in this process. Together, these strategies can mitigate the impacts of climate stress on maize. Collaboration among stakeholders will be crucial for achieving more resilient and sustainable agricultural practices.

The genetic diversity of maize is crucial for addressing the challenges posed by climate change. As global temperatures rise, precipitation patterns become unstable, and extreme weather events increase, crops face mounting stress. Research shows that genetic diversity can enhance maize’s resilience, allowing it to maintain productivity under harsh environmental conditions. In regions like Africa and South Asia, where significant threats to food security exist, there is an urgent need to improve the resilience of maize to ensure the sustainability of food supply [17]. By strengthening the genetic foundation of maize, we can not only address the challenges of climate change but also safeguard global food security. This review aims to investigate research on how the genetic diversity of maize can be utilized for establishing climate-resilient cultivars. This review summarizes the recent scientific advancements, breeding methodologies, and genomic studies to elucidate the potential of genetic diversity as a foundational strategy for protecting maize against climate change. Furthermore, this investigation also evaluates the diverse array of disciplines, including genetics, agronomy, and climatology, to provide a comprehensive understanding of the multifaceted approaches employed to engineer a sustainable future for maize cultivation under increasingly volatile climatic changes.

### 1.1. Genetic Diversity for Climate Resilience

For climate-resilient agriculture, genetic diversity is a cornerstone for developing crops that can adapt to the challenges posed by changing environmental conditions. For maize cultivation, genetic diversity is essential for breeding cultivars with resilience against abiotic stresses such as drought, heat, and soil degradation. Genetic diversity refers to the variety of genetic characteristics of a species or population, comprising allelic variations, gene frequencies, and genomic architectures. Furthermore, genetic diversity is essential for natural selection and artificial breeding, offering various traits that can be used to enhance crop resilience. Maize has a complex genetic makeup and variable phenotypic features. Moreover, it can be utilized for exploring the relationship between genetic diversity and climate resilience. Traditional maize landraces have adapted to diverse agroecological niches over centuries of cultivation and have adaptive traits acquired via natural selection. These landraces have a wide range of morphological, physiological, and biochemical characteristics, which promote resilience against various environmental stresses. Therefore, by focusing on these genetic diversities, breeders can access a diverse array of stress-tolerant traits and incorporate them into novel maize breeding lines.

Recent advancements in genomic technologies have revolutionized the field of genetic diversity in maize, facilitating high-resolution analyses of genome-wide variation and population structure. Furthermore, several studies have comprehensively investigated the genetic architecture of resilience traits and their underlying molecular mechanisms via genome sequencing, single nucleotide polymorphism (SNP) genotyping, and genomic prediction methods. Moreover, quantitative trait loci (QTL) mapping and genome-wide association studies (GWAS) can identify genomic regions related to stress tolerance and prioritize candidate genes for further investigation and breeding efforts. In addition, interdisciplinary approaches integrating genomics, phenomics, and bioinformatics are promising tools to identify the complex association between genotype and phenotype in maize resilience. Phenotypic characterization of diverse maize germplasm under controlled environmental conditions and field trials allows researchers to assess trait performance and identify superior genotypes for breeding programs. Moreover, combining genomic and phenotypic data improves the efficiency and precision of breeding selection processes. This approach accelerates the development of climate-resilient maize cultivars. Genetic diversity is the basis for breeding climate-resilient maize cultivars that can withstand climatic change. Researchers can also evaluate the adaptive potential of maize and secure global food security during uncertain climatic conditions by using the rich genetic variability of maize populations.

### 1.2. Identification of Resilience Traits

In recent years, researchers have focused on identifying maize genetic traits linked to climate resilience, particularly drought tolerance (DT), heat stress (HS) response, and nutrient-use efficiency. These traits are essential for maintaining productivity under increasingly erratic conditions. Comprehensive genomic analyses have clarified their underlying mechanisms. Studies on DT have revealed the genetic basis of root architecture, water transport, and stomatal regulation, identifying candidate genes and regions associated with better water-use efficiency and drought avoidance (Table 1). Analyses of HS response have investigated genes coding for heat shock proteins, antioxidant defenses, and factors that preserve photosynthetic efficiency, shedding light on how maize withstands high temperatures. Research on nutrient-use efficiency has uncovered genetic variation influencing nutrient uptake, assimilation, and remobilization, offering avenues to optimize nutrient utilization. Assessing the genetic architecture of these resilience traits will facilitate the development of climate-resilient maize varieties capable of thriving across diverse environments.

#### 1.2.1. Drought Tolerance (DT)

It has been observed that for the analysis and improvement of crop plants, DT, a most complex quantitative trait, should be assessed [27]. In the last three decades, many studies have been performed to evaluate and increase DT in maize, specifically focusing on predominant and secondary traits such as grain yield (GY) and the anthesis-silking interval (ASI, which is markedly linked with GY), respectively [28]. Reduced ASI can substantially improve GY [29] and has been broadly utilized in conventional breeding for DT. Since the 1990s, several studies have used molecular markers to investigate DT in maize and to elucidate various secondary traits associated with GY under drought conditions in tropical regions [30,31]. Ribaut et al. (1997) [30] indicated different QTLs with small to moderate influence on GY under varying drought stress levels, but these QTLs were generally unstable across different environments. Furthermore, research found that employing a higher marker density in a larger population identified six QTLs for GY under both optimal and drought conditions; however, the overlap of the identified genomic regions across environments was limited [32]. In addition, a study demonstrated that across three populations, there were seven meta-QTLs (mQTLs) and six were expressed in both drought and optimal environments. A meta-analysis of 18 bi-parental populations across various environments identified 15 mQTLs related to GY [33]; however, they were unstable in various environments and genetic backgrounds. Yuan et al. (2019) performed GWAS and identified multiple SNPs and candidate genes for GY under optimal, drought, and heat conditions; however, there were no overlapping SNPs [24]. Overall, the inconsistent phenotypic effects of these QTL across genetic backgrounds suggest that MAS based on them may have limited effectiveness in improving drought tolerance in maize.

#### 1.2.2. Heat Stress (HS)

The literature has also observed that HS has a substantial impact on maize and reduces GY by impairing photosynthetic and reproductive processes [34]. It causes overall growth reduction and leaf scorching, reduces flowering time, enhances ASI, and decreases pollen viability [35]. Although HS does not delay silking with respect to anthesis, it can delay silk receptivity, primarily reducing GY through diminished pollen viability, which influences grain set and kernel number [36]. Early research on the physiological and molecular effects of HS on maize began in controlled environments with seedlings, which later extended to fields and managed HS phenotypes [37,38]. The heat susceptibility index was established by Frey et al. (2016) to characterize segregating families of temperate maize populations [39]. They identified two HS trait-related QTLs on chromosomes 2 and 3. The chromosome 3 QTLs overlapped with regions that were previously associated with pollen viability during HS [40]. Inghelandt et al. (2019) reported QTL for heat susceptibility index traits such as leaf scorching and plant height on chromosome 9 [41]. Moreover, a collaborative GWAS study was conducted by Purdue University, CIMMYT, and National Agricultural Research System (NARS) partners from South Asia on test crosses of >500 diverse maize lines for GY and related traits during HS. This investigation revealed five haplotype blocks and eight SNP variants for GY. In addition, CIMMYT confirmed the presence of 22 GWAS-identified genomic regions for GY or related traits under HS in the independent biparental Asian population. Moreover, three major QTL intervals affecting various HS-related traits were identified from multiple population analyses. Additionally, CIMMYT’s GWAS panel investigated the lipid traits in both heat-stressed and non-stressed environments and found 78 significant SNP correlations at 40 genetic loci and 53 candidate genes. Overall, CIMMYT and partners revealed that in maize, GY under HS or heat + drought stress is polygenic. Therefore, the most effective breeding method to improve GY under these stresses is the whole-genome prediction.

#### 1.2.3. Waterlogging Tolerance (WT)

In maize, WT is associated with complex genetic and physiological responses. Early temperate maize investigations have indicated that traits related to WT in seedlings have complex inheritance and expression [42,43]. These responses include anaerobic protein synthesis, metabolic shifts to fermentative pathways, gene expression alterations, and structural modifications like the formation of aerenchyma. A CIMMYT research on tropical maize lines found that 7 days of waterlogging at the V7–V8 stage had polygenic WT inheritance with partial dominance of tolerance over susceptibility, suggesting that additive variance had a more significant role than non-additive variance in GY modulation during waterlogging conditions [44]. A QTL analysis of F_2_ families from a biparental population revealed various QTLs for WT, which accounted for 3.9 to 37.3% of phenotypic variance [45]. Moreover, various QTLs were linked with plant height as well as shoot dry, root dry, and total dry weights. In addition, their WT coefficients were mapped on chromosomes 4 and 9. Mano et al. (2016) observed that in teosinte (wild maize progenitor) [46], large-effect QTLs affected the morphological modifications including adventitious root development and the formation of aerenchyma. Zaidi et al. (2010) investigated the GY and related secondary traits of the waterlogging-tolerant recombinant inbred line (RIL) and elite susceptible CIMMYT lines during waterlogging stress at the V7–V8 stage [44]. They revealed six QTLs on chromosomes 1, 2, 3, 5, 7, and 10, which explained about 40% of the phenotypic variance. Moreover, it was observed that the GY and the brace root QTL on chromosomes 1 and 7, respectively, were co-localized with previously identified QTLs for aerenchyma formation [47] and caused teosinte accession Zea nicaraguensis. Therefore, based on the polygenic nature of WT, the most efficient breeding technique to improve GY performance under waterlogging stress is the whole-genome prediction method.

#### 1.2.4. Cold Tolerance (CT)

The CT in maize is polygenic, involving dominance, additive, and maternal effects [48]. Over the past two decades, studies on temperate maize have identified numerous QTLs affecting traits like early seedling vigor [49,50], germination ability [51], early growth, and chlorophyll fluorescence [52]. Although most investigations are focused on seedling-stage chilling tolerance, only a few studies have been performed on cold stress during the flowering or vegetative stages. CIMMYT performed GWAS on 306 tropical testcross hybrids during field conditions at vegetative, seedling, pre-flowering, and flowering stages. They found 29 SNPs with substantial effect, which were common in these stages for CT. Furthermore, various significant SNPs clustered at 2.08, 6.01, and 10.04 chromosomal bins, regions that were previously related to cold stress traits [53]. Because of the minimal impact of these QTLs, MAS might be effective for improving CT in tropical maize. Therefore, whole-genome prediction is recommended as the most suitable breeding method.

### 1.3. Disease Resistance in Maize Under Changing Climates

Climatic changes are increasing the prevalence and impact of maize ear and stalk rots, specifically those caused by Fusarium spp. The inheritance of Fusarium ear rot resistance is complicated. Furthermore, hybrids have been observed to have 27% and 30% less ear rot and fumonisin content, respectively, than their inbred parents in diallel mating studies [54]. CIMMYT has indicated multiple elite lines with reduced fumonisin accumulation [55]. Moreover, QTL mapping investigations have indicated that Fusarium ear rot resistance is a quantitative trait modulated by polygenes with minimal impacts [56]. However, moderate-effect QTLs have been observed on chromosomes 3 and 4 [57,58,59]. GWAS has demonstrated SNPs related to fumonisin content and Fusarium ear rot, and in biparental populations, some of these were found near QTLs [56,60]. Genetic mapping for Gibberella stalk rot resistance identified two QTLs, qRfg1 and qRfg2, which increase resistance by 32–43% and about 125%, respectively. These QTLs have been fine-mapped to ~500 and ~300 kb regions on chromosomes 10 and 1, respectively [61]. Moreover, functional genomic analyses revealed that qRfg1 promoted resistance via induced and constitutive enhanced expression of defense-related genes, whereas qRfg2 increased the resistance by reducing the auxin signaling induction and polar auxin transport [62]. In addition, another QTL, Rgsr8.1, induced wide-spectrum resistance against Gibberella stalk rot on chromosome 8 [63]. A GWAS study of Asia-adapted CIMMYT maize lines indicated that multiple SNPs were linked with Charcoal rot and Fusarium ear rot in different chromosomes. For each stalk rot, two mapping populations were established with a common susceptible parent. These GWAS investigations identified two QTLs on chromosomes 6 and 8 for charcoal rot resistance and one QTL on chromosome 8 for Fusarium ear rot resistance, validating their significance in disease-resistance breeding. 

### 1.4. Association Studies for QTL Identification and Candidate Gene Mining

#### Genome-Wide Association Studies

**Figure 1:**
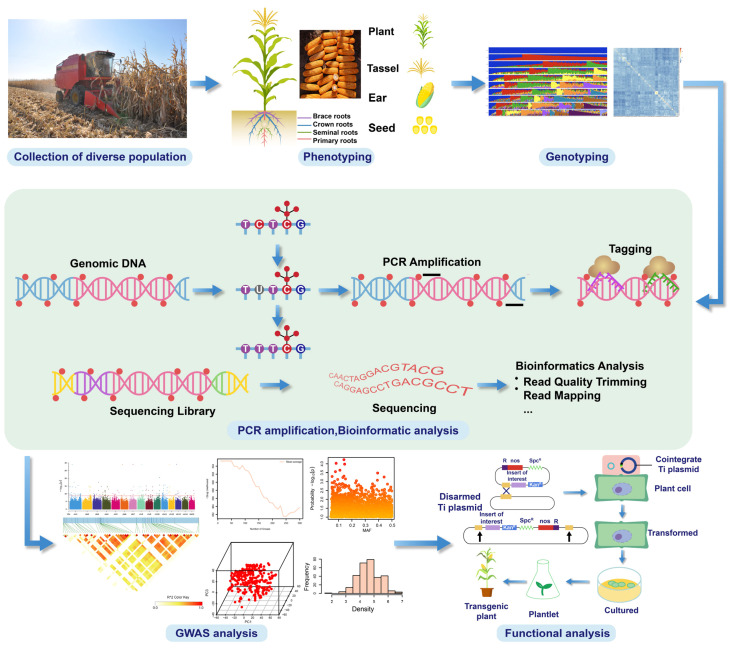
An illustration of the selection of diverse germplasm, followed by resequencing, for genome-wide association studies in maize for the evaluation of candidate genes and key QTLs for trait improvement.

GWAS has been substantially employed for the identification of candidate genes and genomic regions linked with different resilience traits in maize, thereby opening doors for MAS in breeding programs. GWAS, a robust method, employs the genotypic and phenotypic variations of plant species to identify alleles related to desirable traits. Furthermore, GWAS have been crucial for investigating the connection between genetic markers and traits in maize research (Figure 1). The inaugural maize GWAS in 2008 was focused on identifying SNPs that influenced the oleic acid content in kernels via 8590 loci in 553 elite inbred lines [64]. After the “B73” reference genome was published, GWAS rapidly gained prominence and is now a standard approach for elucidating genotype-phenotype relationships in maize, effectively identifying genes’ functional genomics [65,66]. Studies have identified genes responsive to abiotic and biotic stresses, providing critical insights and enhancing our understanding of maize’s genetic structure [67]. In addition, GWAS have also facilitated precise QTL mapping within a diverse maize population, assessing numerous historical recombination events, which leads to rapid linkage disequilibrium decay, achieving mapping accuracy at the single-gene level (Table 2) [68].

Maize GWAS are distinguished by their rapid linkage disequilibrium decay, which is due to the crop’s diverse phenotypic and genetic characteristics [110]. GWAS have successfully identified QTL and genomic regions responsible for disease tolerance in maize, such as resistance to leaf necrosis, various ear rots, and southern maize rust, offering a quicker method to identify the genetic basis of complex traits compared to the traditional linkage mapping [67]. Additionally, GWAS have proven effective in identifying regions related to abiotic stress, highlighting genetic diversity for physiological and morphological traits in maize populations [111,112]. Maize is an ideal crop for GWAS due to its advanced genotyping technologies and extensive marker density, which allow the investigation of natural variation and detailed quantitative trait mapping. Furthermore, high-resolution genotyping technologies, such as the IlluminaTM 9k SNP chip, promote GWAS for the identification of novel alleles that can increase maize adaptability and productivity [113,114].

### 1.5. Gene Editing Technologies

#### 1.5.1. Gene Editing Unveils Key Regulators in Maize Development

**Figure 2:**
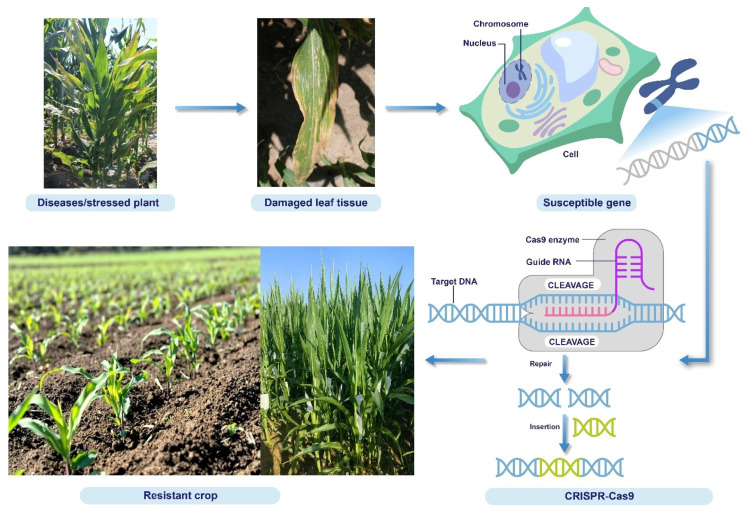
A schematic of the CRISPR technology employed for the development of new maize germplasms and functional genomics analysis.

Gene editing has revolutionized our ability to decipher gene functions and establish new crop varieties in maize (Figure 2) [115,116]. The CRISPR technology has enabled researchers to explore the roles of numerous genes associated with maize development, growth, and stress responses. Maize production is severely impacted by various abiotic stresses, such as flooding, extreme temperatures, drought, and adverse soil conditions. Furthermore, traditional breeding methods have limitations in developing stress-tolerant varieties; therefore, genome editing technologies such as CRISPR are promising alternatives. Although traditional transgenic technology has been employed to overexpress specific genes for the verification of gene functions and the development of crops with improved traits, its widespread application has significant challenges. Moreover, multi-gene stacking and strict regulations governing genetically modified crops are difficult. The gene activation technology based on CRISPR is an essential tool for the simultaneous activation of multiple genes, overcoming the limitations of traditional transgenic approaches. This technology provides a new strategy for the improvement of crop genetics via key genes that positively modulate particular agronomic traits [117].

Gene editing studies have identified several genes as crucial regulators of maize growth and development. *ZmSMC3* is crucial for meiotic centromere pairing and cohesion of sister chromatids. Furthermore, its knockdown resulted in reduced growth and dwarfism [118]. *MMS21* has been observed to be associated with pollen germination, vegetative and root growth, and seed development by modulating genome function and stability [119]. Moreover, *ZmNRPC2* modulates RNA polymerase III activity and the levels of genes related to kernel development, whereas its knockdown markedly reduced kernel size [120]. *ZmThx20* has been observed to regulate kernel size and the accumulation of storage protein in seeds, whereas its mutation can shrink the kernels [121]. *AGAP* and *KNR6* are necessary for vesicle trafficking, and their mutations can cause severe defects in roots, dwarfism, inflorescences, and short ears with fewer kernels [121]. CC-type glutaredoxins such as *ZmGRX2*, *MSCA1*, and *ZmGRX5* induce the target protein’s redox status, and their triple knockout mutants lead to significantly suppressed tassel and ear growth, as well as dwarfism [122]. *YIGE1* modulates ear length and inflorescence meristem size by regulating auxin and sugar signaling pathways [123]. *ZmMIC1* has also been found to affect nicotianamine biosynthesis and 5′-methylthioadenosine salvage, thereby affecting seedling growth [124]. Furthermore, *ZmPAT7* and *ZmPT7* are crucial for tassel branch number and inorganic phosphate acquisition, respectively [125]. Through gene editing, researchers have uncovered comprehensive data on the genetic mechanisms underlying maize growth, development, and yield-related traits, providing a basis for the establishment of improved maize varieties.

Environmental stresses cause stalk lodging, which is a significant threat to maize quality and yield. Therefore, developing maize lines with increased stalk strength is essential to ensure high and stable yields. Researchers have successfully edited the stiff1 gene, a negative stalk strength modulator, via CRISPR. The edited allele with a 2 bp deletion and frameshift mutation conferred stronger stalks in the edited plants, which allowed enhanced-density planting and prevented lodging [118].

Furthermore, researchers have also generated Semidwarf maize plants by editing the *ZmGA20ox3* gene via the CRISPR technology. These varieties are suitable for high-density planting and have improved lodging resistance, indicating the development of new germplasm with enhanced stress tolerance [118].

One of the predominant limiting factors for maize production is drought stress. Recently, scientists edited the promoter sequence of the *ARGOS8* gene to upregulate its expression, which resulted in enhanced GY under drought conditions [124]. CRISPR has, therefore, become a hotspot technology for the establishment of novel germplasm sources for breeding stress-tolerant maize varieties. By precisely editing key genes and regulatory regions, researchers can develop maize lines with improved tolerance to various abiotic stresses, ensuring stable and high yields under challenging environmental conditions.

Scientists have also successfully established a CRISPRd/cas-based toolkit for precise and effective activation of specific target genes in maize [126]. Moreover, this toolkit allows precise control over gene expression levels by targeting specific promoters or regulatory regions without introducing foreign DNA into the plant’s genome. This technology can develop improved maize varieties with enhanced performance and desirable characteristics by activating endogenous genes involved in various agronomic traits, such as growth, yield, development, and stress tolerance. The CRISPR-based gene activation approach offers a promising strategy for crop genetic improvement, circumventing the challenges associated with traditional transgenic methods while enabling precise and multiplex gene regulation. In addition, CRISPR technology empowers researchers to utilize the plant’s genetic material, providing a more efficient and sustainable method for crop improvement.

#### 1.5.2. Virus-Induced Gene Silencing (VIGS)

VIGS is a powerful technology that utilizes a plant’s natural antiviral defense mechanism, which is mediated by RNA [127,128]. When a plant is infected with unmodified viruses, this mechanism targets the viral genome. However, researchers have adapted this mechanism for functional genomics studies by introducing virus vectors carrying inserts derived from host plant genes [129]. In this case, the RNA-mediated silencing process is redirected to target the corresponding mRNAs of the host genes, effectively knocking down their expression. VIGS has been broadly utilized for assessing gene functions and has proven to be a valuable tool for high-throughput functional genomics studies. Furthermore, VIGS allows rapid and efficient target gene silencing, enabling researchers to study their functions in a relatively short time frame. Moreover, it can be applied to various plant species, including those that are difficult to transform or regenerate from tissue culture. VIGS can be adapted for high-throughput screening of gene functions, facilitating large-scale functional genomics studies. In addition, unlike stable genetic modifications, VIGS promotes transient silencing of target genes, which allows researchers to investigate gene functions without permanently altering the plant’s genome.

Several genes have been silenced via VIGS in maize. Cao et al. (2021) [130] employed VIGS to investigate the role of *ZmbZIP33* in maize. Silencing *ZmbZIP33* expression (*BMV-ZmbZIP33*) reduced chlorophyll levels and abscisic acid (ABA) levels, decreased peroxidase and superoxide dismutase activities, as well as enhanced water loss rate and stomatal opening compared to the BMV control. These findings indicated that silencing *ZmbZIP33* can reduce drought resistance and impair maize plants’ recovery ability. Batool et al. (2024) utilized VIGS to investigate the function of the *ZmWRKY36* gene in maize and revealed that silencing ZmWRKY36 can reduce chitin-triggered reactive oxygen species (ROS) production [16]. This finding validates the positive regulatory role of *zmWRKY36* in the maize immune response. VIGS utilizes plants’ natural antiviral defense mechanisms and has, therefore, become an invaluable tool for functional genomics research, enabling scientists to elucidate the roles of specific genes in plant development, growth, and stress responses. VIGS has increased our knowledge of gene functions and paved the way for more targeted crop improvement strategies.

### 1.6. Breeding Strategies for Climate Adaptation

#### 1.6.1. Utilization of Maize Wild Relatives as a Genetic Resource

Maize is a member of the family Poaceae, which comprises seven genera: *Tripsacum*, *Sclerachne*, *Polytoca*, *Trilobachne*, *Chionachne*, *Coix*, and *Zea*. The genus *Zea* comprises four species, of which only *Zea mays* L. (2n = 20) has economic importance. Teosintes, another *Zea* species, are wild grasses that are native to Central America and Mexico [131]. Maize (*Zea mays* L.) was identified as originating > 7000 years ago and potentially evolving from annual teosinte (*Zea mexicana*) via gradual selection processes. Modern maize and its wild ancestor have similar plant growth and architecture. However, *Tripsacum* species have higher chromosome numbers (2n = 36, 64, or 72), complicating hybridization with maize. These wild ancestors harbor substantial genetic diversity, offering a valuable resource for germplasm enhancement. Molecular evolution investigations on maize, for instance, have employed genomic and genetic tools established for *Tripsacum* [132]. Orthologous genes analysis in *Tripsacum* and maize identified specific genes in *Tripsacum* with frequent non-synonymous substitutions. Furthermore, these genes were frequently observed during the adaptation of domesticated maize to temperate regions via artificial selection. Additionally, Blakey et al. (2007) [132] found that phospholipid metabolism, an intermediate metabolic pathway, was related to freezing and cold tolerance. Moreover, anatomical characteristics of *Tripsacum* like aerenchyma tissue in roots and other traits promote drought stress resilience. Studies on *Tripsacum* investigation root penetration and increased biomass have further highlighted its drought resistance. In addition, physiological analyses revealed that *Tripsacum’s* exceptional drought resistance was attributed to its high photosynthetic rates and water use efficiency [133]. Subsequent studies demonstrated that maize cultivars introgressed with *Tripsacum* grow better under drought stress than modern maize. These introgression lines indicated superior root systems that penetrated deeper into the soil and had higher GY than contemporary maize cultivars [134]. The investigation of hybrid calli from maize *Tripsacum dactyloides* L. (eastern gamagrass) × (*Zea mays* ssp. mays) revealed that they had greater fresh weight during salt stress, indicating enhanced salt tolerance [135]. These hybrids could retain sodium in their leaves, which reduced water potential and maintained turgor pressure, crucial for vegetative growth [136]. Therefore, utilizing the genetic potential of wild relatives is essential for the development of novel genetic resources, which can be used to create maize lines with improved tolerance to abiotic stresses.

#### 1.6.2. Speed Breeding in Maize

Traditional maize breeding involves developing high-yielding hybrids through selective mating of parent lines based on their combining abilities. This is a prolonged and intricate breeding process, as it usually takes 4–6 years to develop parental lines and an additional 1–2 years for their hybrids. To expedite this process, novel rapid breeding techniques have been proposed [137,138]. Different strategies can be employed to shorten the maize breeding cycle, including off-season nurseries. Furthermore, growing crops in different locations that are better suited to their photoperiodism can accelerate breeding cycles. This method allows for multiple generations per year; however, it requires quarantine clearance, other permissions, and time for transporting genetic material, as well as has a risk of new pest infections and diseases. Double haploid (DH) is a novel technology that can develop homozygous lines in just two generations and, thus, substantially reduce the time of the breeding cycle [138]. The most frequently employed method for producing DH maize includes the use of the R1-nj color marker. However, despite its efficiency, DH technology has low success rates in tissue culture adaptation and haploid induction. The doubling process is a genotypic-dependent method, which involves the use of colchicine, a carcinogenic agent. Therefore, DH technology (homozygosity per generation) should be combined with off-season nurseries (multiple generations per year) to breed homozygous and homogenous lines. Each of these methods has advantages and disadvantages, but with their implementation, the overall breeding cycle for maize can be substantially shortened, facilitating the development of new hybrids and improving breeding efficiency.

#### 1.6.3. Enhancing Maize Breeding with Genomic Selection and Speed Breeding

**Figure 3:**
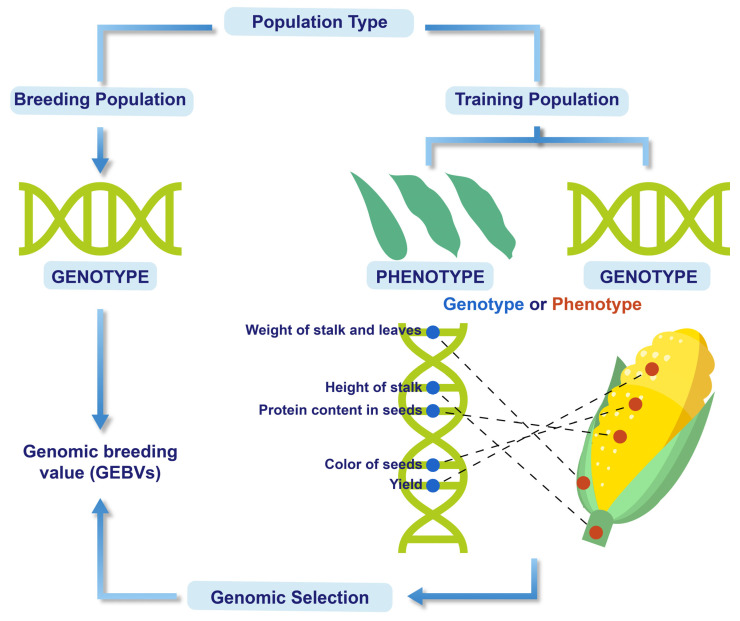
The illustration of steps involved in the genomic selection process for improving maize varieties.

MAS is only feasible if the target trait is linked with one or very few major genes. Furthermore, it is irrelevant or impractical for quantitative traits governed by multiple minor genes [139]. Most stress tolerance-related traits fall into this category. To overcome this limitation, a new selection tool called genomic selection (GS) was introduced, which selects such traits by elucidating an individual’s net genetic merit by assessing the effects of dense markers distributed genome-wide (Figure 3) [140]. In GS, the effect of each marker is assessed, and the sum of all marker influences is utilized to elucidate the genomic estimated breeding value (GEBV) of each individual. The GS has proved to be an effective tool in improving polygenic traits, including plant yield, modulated by various small-effect genetic loci. This approach utilizes marker effects across the genome to acquire GEBVs. Studies have shown that GS can produce higher stover and GY compared to MAS in bi-parental maize populations [141]. Moreover, in multi-parental maize populations, conducting two fast breeding cycles per year via GS has been observed to increase the GY by approximately 2% [142]. The genomic prediction accuracy is closely linked to the correlation between marker-predicted phenotypic and genotypic values in validation populations. GS has demonstrated higher prediction accuracy than both traditional GS and MAS. This method can effectively identify alleles with smaller additive influence, thereby increasing genetic gains during drought conditions. However, the predicted data can be slightly different between validation and training sets or due to linkage disequilibrium with causal variants [143].

Combining GS with MAS (GS-MAS) has indicated better efficiency than the QTL-MAS approach for drought-stressed growth traits including anthesis, GY, and plant height. This can be observed from variabilities in prediction accuracies (GS-MAS) and R2 (QTL-MAS) [144]. Furthermore, employing the phenotypic and molecular marker data as input variables provides higher-quality estimates for GY than with phenotypic data alone [145]. It has been observed that maize is extremely vulnerable to abiotic stresses such as waterlogging and drought, which results in a high degree of environmental × genotype interaction within breeding programs. To identify hybrids and lines with increased yield potential, targeted or managed stress conditions are utilized. However, due to poor field performance or the low genetic value of selected parents, many hybrids, parents, and crosses are often discarded. Therefore, for crossing, parents with high GEBVs should be utilized for acquiring maximum favorable alleles for desirable traits in progenies. High environmental error often limits genetic gain under abiotic stresses [145]. Integrating GS with speed breeding techniques for abiotic stress tolerance can enhance the accuracy of parent selection, thereby increasing genetic gain and improving abiotic stress resilience. Speed breeding methods, such as double haploid (DH) technology, off-season nurseries, and in vitro nurseries, can substantially shorten breeding cycles and promote the rapid development of novel stress-tolerant maize varieties. By utilizing the strengths of both GS and speed breeding, maize breeding programs can achieve more efficient selection and faster establishment of stress-tolerant, high-yielding hybrids.

#### 1.6.4. Marker-Assisted Selection (MAS)

In the past, crop selection primarily relied on the identification of phenotypic characteristics of crop germplasm under field conditions. These traits were used to grow new varieties via conventional breeding. However, this method has significant drawbacks, as the environment can influence phenotypic traits, which may result in the selection of suboptimal breeding materials. Furthermore, it can limit the germplasm’s gene pool, thereby reducing the effectiveness of crop development. Despite the significant contributions of traditional breeding to agriculture over the past several decades, it is time-consuming, labor-intensive, environmentally dependent, and often inefficient [146]. Therefore, researchers are investigating novel alternatives for sustainable agriculture crop improvement. The development in breeding technology, specifically MAS, has markedly enhanced breeding efficiency. Moreover, molecular markers are not influenced by environmental conditions like phenotypic traits, and provide consistent precision throughout the selection process, making MAS a highly attractive breeding method. In addition, MAS can precisely track the genes responsible for desirable traits within the crop genome, facilitating accurate breeding [147]. Modern crop enhancement is predominantly achieved via mutation breeding, cross-breeding, and transgenic breeding. Over the years, plant breeding has evolved through different methods, such as hybrid breeding, artificial selection, precision breeding, and molecular breeding. Initially, plant breeding involved the selection of superior plant phenotypes from early hybrids during domestication. However, Mendel’s discovery of genetic laws and the further knowledge of genetic structures indicated the importance of genotypes to phenotypes, formulation of the central dogma, and development of hybrid varieties and mutation breeding. Furthermore, plant breeding was revolutionized with the advent of molecular technology, which resulted in the establishment of molecular plant breeding, which involved MAS of domesticated plants and their wild relatives. In the last two decades, substantial progress in next-generation sequencing technology has developed genomics-assisted breeding, a more efficient alternative to conventional breeding for complex traits. Genomics has markedly improved selection precision and intensity, providing genetic gains in crop development while notably decreasing labor requirements and breeding cycle time. Recently, CRISPR genome editing in plant breeding has emerged as a promising approach for improving various crop traits related to quality, yield, and stress tolerance [148,149]. CRISPR allows plant breeders precise control over targeted genetic modifications, offering a transformative resource for rapid agricultural crop improvement [150]. Moreover, constant development in CRISPR systems, including base editing tools and CRISPR/Cpf1, have made genome editing a cost-effective, widely accepted, and easy-to-use technique for targeted genetic modifications in numerous crops.

Traditional MAS employing QTL-MAS has been utilized for increasing the speed and efficiency of maize breeding programs [151,152,153]. Numerous QTLs have been found for different traits, including GY in well-watered [154,155] and drought-stressed conditions [156,157], as well as normalized difference vegetation index (NDVI) and plant height [158], root traits [32], and stay-green [156]. However, for MAS to be effective, it is crucial to identify QTLs that are consistent across different environments and populations [159]. Because of the genotype-by-environment interactions, the assessed genetic association of traits with QTLs often varies between environments [160,161]. Additionally, for a particular trait, QTLs can differ based on the genetic background between inbred lines and their testcross hybrids [162,163]. In the past, QTL studies had limited breeding utility, as genetic resolution and marker densities were often too low. Recently, genotyping-by-sequencing (GBS) has been established to enhance molecular markers availability from around 100 to thousands of SNPs evenly distributed in the genome [164,165]. This advancement has reduced the confidence interval surrounding QTLs, enabling the establishment of high-resolution genetic maps and precise QTL mapping.

### 1.7. Agronomic Practices for Climate Mitigation in Maize

**Figure 4:**
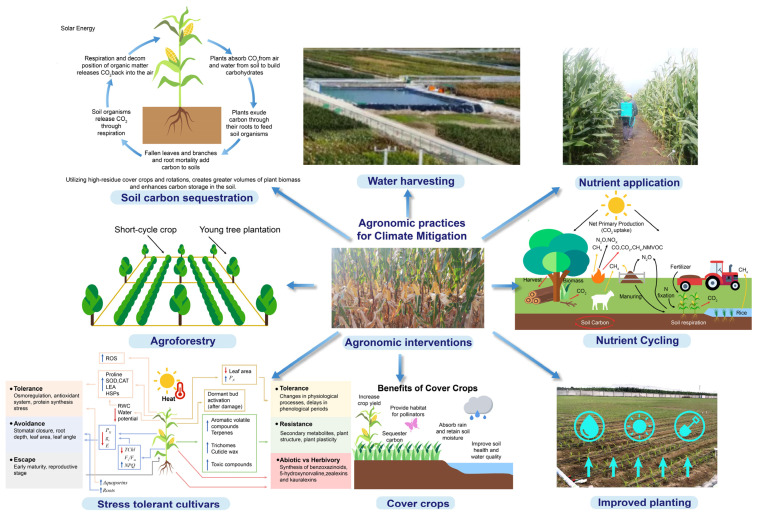
A comprehensive overview of various agronomic practices implemented to mitigate the influence of climatic change on maize cultivation, thereby increasing the crop’s resilience against the climate.

Climate change has been a constant phenomenon throughout history; however, it has been rapidly accelerating due to human activities and has become one of the most pressing issues globally, profoundly affecting agriculture [166,167]. Changed precipitation patterns, increasing temperatures, and severe weather events are negatively impacting crop yields and soil health, thereby posing a threat to food security and the livelihoods of millions. Agronomy, a field that focuses on crop production and soil management, has played a crucial role in addressing these challenges (Figure 4) [168]. Practices that enhance soil health, conserve water, and minimize greenhouse gas emissions should be implemented by the farmers to build resilience against climate change and support global emission reduction efforts. The widespread impacts of climate change on agriculture, including temperature rises, shifts in rainfall, and extreme weather, have jeopardized food security and the well-being of populations worldwide [169]. Therefore, agronomic management practices are essential for alleviating the effects of climate change. Although agriculture is a primary source of greenhouse gas emissions, it can also capture carbon and lower emissions [169].

#### 1.7.1. Conservation Agriculture

This approach involves minimizing or eliminating tillage to preserve soil moisture, reduce soil erosion, and enhance soil organic matter. Furthermore, it conserves energy by reducing the need for tillage and reduces fossil fuel consumption as well as associated emissions from soil disturbance [170]. Moreover, conservation agriculture can increase soil carbon storage, contributing to the reduction of greenhouse gas emissions.

#### 1.7.2. Precision Farming Technologies

These technologies include GIS mapping, remote sensing, and variable rate technology, which are employed to optimize resource use efficiency and mitigate climate risks in maize cultivation. Furthermore, these advanced technologies allow the precise application of inputs such as fertilizers and water, thereby minimizing the environmental impact of agriculture [171]. Therefore, farmers can reduce input usage, boost crop yields, and decrease emissions from agricultural activities by adopting precision agriculture practices.

#### 1.7.3. Climate-Smart Crop Management

Climate-resilient crop management includes practices like crop rotation, intercropping, and agroforestry, which enhance resilience, diversify income streams, and promote ecosystem services in maize agroecosystems. Moreover, this approach promotes sustainable alterations in cropping patterns to acquire more resilient and diverse agricultural systems [172]. Planting a variety of crops can reduce pests and disease pressure, improve soil health, and provide diverse food and income sources. In addition, increased crop diversity also reduces the risk of crop failure due to extreme weather events like floods and droughts.

#### 1.7.4. Agroforestry Integration

Agroforestry integrates trees into agricultural landscapes to improve soil health, provide shade for crops, and sequester carbon. Furthermore, it provides multiple ecosystem services, such as enhancing soil fertility, reducing erosion, and providing wildlife habitats. [173]. Moreover, agroforestry integrates carbon in woody biomass and soil organic matter, which mitigates greenhouse gas emissions [174].

#### 1.7.5. Cover Cropping Benefits

Cover cropping involves planting crops primarily to enhance soil health and protect against erosion. These crops increase soil organic matter, improve soil structure, and reduce the need for tillage. Cover cropping enhances soil health and prevents erosion, thereby mitigating the influence of climate change [175,176].

#### 1.7.6. Policy Support for Sustainable Agriculture

Governments can support sustainable agriculture practices through subsidies for conservation agriculture, agroforestry, and crop diversification. Policies that promote renewable energy use, such as wind and solar power, can also reduce greenhouse gas emissions and mitigate climate change impacts on agriculture [171].

### 1.8. Technological Innovations for Resilience Enhancement

#### 1.8.1. High-Throughput Phenotyping (HTP)

Conventional phenotyping for assessing drought, heat, and salt tolerance in maize typically employs destructive methods, limiting breeding programs. The advent of HTP platforms has revolutionized this field by significantly reducing these limitations. Researchers have employed different HTP methods to characterize maize phenomes, such as electrical conductivity sensors for soil variability mapping, thermal imaging for plant canopies, penetrometers, and spectral reflectance [177]. Additionally, mobile cameras, satellite imagery, ground-based platforms, and UAV imaging have been significantly utilized for stress assessment in maize. Furthermore, HTP is often achieved through photographic data collection, which quantifies characteristics of a plant’s life cycle, thereby resolving the limitations of expenses, inefficiencies, and inaccuracies of traditional methods [178]. Although powerful, satellite imaging is less commonly applied for the large-scale agriculture of small experimental plots; however, it has shown strong correlations with UAS multispectral imagery in maize plots [179]. Furthermore, the thermal imaging-acquired UAV-RGB images allows precise isolation of maize canopy temperature [180]. Moreover, advanced tools such as stable isotopes, digital imagery, and thermal imagery have increased accuracy for collecting soil and climate data. Researchers have employed NDVI, SPAD meters, and infrared gas analyzers to quantify the impact of the environment, digital imagery to assess early biomass, and thermal imagery for leaf temperature quantification during transpiration [181,182,183]. Techniques like spectroscopy, infrared thermography, and fluorescence imaging have been used to monitor responses to stress and scrutinize plant growth [184,185]. In addition, multispectral imaging techniques such as MRI, ultraviolet spectra, and X-ray CT can indicate an alteration in stomatal conductance and ionic balance, which causes drought and HS resistance in maize [128]. The rapid advancements of HTP have produced extensive datasets, posing analytical challenges. Emerging deep learning (DL) and machine learning (ML) techniques offer promising solutions for identifying the underlying correlation in large datasets, facilitating the real-time HTP of plant traits [178,186,187]. ML models must be robust and flexible to handle multiple stress and disease symptoms on single leaves or canopies, with training data continuously updated to reflect stress symptom complexity.

#### 1.8.2. Omics-Based Approaches for Developing Climate-Resilient Maize

The development of climate-resilient maize includes the use of various omics-based approaches, such as genomics, transcriptomics, proteomics, metabolomics, and microbiomics. These approaches help identify key genes, proteins, metabolites, and microbial communities that contribute to stress tolerance and adaptation.

#### 1.8.3. Transcriptomics

Genomic prediction, or the ability to predict traits using genome-wide sequence data, has substantially revolutionized breeding methodologies and enhanced our comprehension of complex traits’ genetic foundations. Moreover, transcriptome data may augment the accuracy of genomic predictions. In biology, it is difficult to predict complex traits from genetic data; however, successful predictions are significantly valuable for animal and plant breeding programs [188,189]. The GS or prediction utilizes all available genetic indices to surpass the limitations of MAS, which is solely focused on significant QTLs and is less effective for traits governed by numerous small-effect alleles [152,190]. This comprehensive approach enables breeders to make data-driven, informed decisions about which lines should be utilized for the breeding programs, thereby accelerating development and reducing costs for new crop varieties [191,192]. Furthermore, genomic prediction models associate phenotypes with genetic signatures, highlighting complex traits’ genetic architecture. However, consistent with GWAS and QTL mapping, the translation of associated genetic markers into the molecular mechanisms underlying traits remains challenging [193,194]. A promising solution is integrating additional omics data with genetic variation to consider intermediate biological processes. This integrative approach has shown promising, yet sometimes mixed, results in plants. For instance, transcriptional data of parental lines have been used for predicting hybrid performance. Measures of transcript level-based distances for traits have outperformed genetic markers for predicting the performance of hybrid maize [195,196]. However, the use of all transcripts instead of a selected subset reduced the model performance [197]. The efficacy of transcript-based models compared to genetic marker-based models varies depending on the trait. In addition, the transcriptome data better predicted GY, while genetic indices more effectively predicted the grain dry matter weight in hybrid maize populations [198]. Consistently, for specific traits in a maize diversity panel, models comprising both marker and transcript data outperformed marker-only models [199]. Therefore, additional omics data should be incorporated for trait prediction in *Drosophila melanogaster* [200] and human diseases like breast cancer [200], as well as treatment responses such as acute kidney rejection and infliximab in ulcerative colitis [201,202], to highlight the efficiency of transcriptome data in precision medicine. Overall, these data indicate that transcriptome data can enhance the accuracy of trait prediction models.

##### Metabolomics

Maize research has greatly advanced through the application of omics techniques, contributing significantly to our understanding of its systems biology. Since maize has a dual role of bioethanol source and staple food, its metabolism has been extensively studied. Medeiros et al. (2021) reviewed different key factors of maize metabolism, such as basic molecular processes, responses to abiotic and biotic stresses, and beneficial biotic interactions, thereby highlighting the critical functions of the maize metabolome [203]. Furthermore, studies have investigated maize kernel’s nutritional content and the molecular mechanisms that mediate the production of specific metabolites. Moreover, how metabolic models and the metabolome correlate with leaf physiology has also been studied. In addition, the genetic modification-induced metabolic alterations in maize have been identified. The degree of natural metabolic variability in maize and its potential application in breeding programs have also been explored. Alvarez et al. (2008) highlighted the significance of root-to-shoot signaling under drought conditions and observed the changes in stomatal hormones such as ABA and cytokinins, as well as an increase in intermediates of the phenylpropanoid pathway [204]. Casati et al. (2011) utilized metabolomics to evaluate myoinositol as a candidate signaling molecule in UV-B responses during the temporal irradiation of maize canopy leaves [205]. Furthermore, Witt et al. (2012) investigated the metabolome alterations under drought [206]; however, they did not find any significant differential changes, which might be because their experimental design involved a greenhouse setup instead of field conditions. Richter et al. (2015) conducted metabolite profiling under salt stress and found that the TCA cycle and sugar metabolism were significantly affected in two differently resistant maize hybrids [207]. Moreover, Sun et al. (2016) [208] evaluated maize’s metabolic response to cyclic drought and revealed that different metabolic pathways returned to normal at varying speeds during recovery. Their study also revealed quantitative differences between drought cycles, underscoring the complexity of metabolic processes triggered by water cycle changes. In addition, metabolomic studies have also identified the maize organs most significantly affected by abiotic stress. For instance, during drought, the highest metabolic change is observed in leaf blades [206,209], whereas during high-salt conditions, shoots undergo greater metabolic alterations than roots [210]. Ganie et al. (2015) indicated that under phosphorus starvation, leaves are the primary site of metabolic changes [211]. These findings illustrate that metabolomics can reveal the intricate metabolic networks in maize and their responses to various stresses. This knowledge is crucial for developing strategies to enhance maize resilience and productivity under challenging environmental conditions.

### 1.9. Integrated Modeling for Climate-Resilient Maize Varieties

Climate change poses a significant threat to global food security, particularly affecting maize, a staple crop for millions of people. Therefore, the development of climate-resilient maize varieties is significant for mitigating climatic change’s effect on crop yields and ensuring food security. This can be achieved by integrated modeling approaches, which are powerful tools that combine crop models, climate models, and various other data sources. Key components of integrated modeling for climate-resilient maize typically include crop modeling, climate projections, genetic information, and advanced data analysis techniques. Crop models can simulate maize development, growth, and yield under different environmental conditions based on factors such as precipitation, temperature, soil characteristics, and management practices [212]. Climate models provide future scenarios of temperature, precipitation, and extreme weather events, which are essential for understanding the potential impacts of climate change on maize production. Furthermore, genetic information allows researchers to evaluate and select traits that modulate climate resilience, such as DT and heat resistance [35]. Integrated modeling approaches have several benefits in developing climate-resilient maize. These models enable breeders to develop maize varieties with specific traits suited to future climate conditions. For example, crop modeling has indicated that climate-resilient varieties can provide 5–25% higher yield in many maize-growing areas of eastern and southern Africa [212]. The future scenario simulation allows researchers and policymakers to identify regions where current maize varieties may become unsuitable and plan for the introduction of climate-resilient alternatives [35]. Moreover, integrated models also help optimize resource use, such as water and fertilizer, by predicting crop responses to different management practices under changing climate conditions [213].

Several studies have indicated the effectiveness of these approaches. For instance, a novel hybrid modeling approach integrated the CERES-Maize crop model with ML techniques to elucidate the impact of climate change on the productivity of different hybrid maize in China. Their findings revealed that climate change would predominantly have adverse impacts on maize yields, with the extent of yield reductions heavily influenced by the growth cycle characteristics of the hybrids. Although introducing new hybrids can partially mitigate the yield loss caused by climate change, if no adaptation measures are taken, approximately 53% of the cultivated areas would necessitate hybrid replacements before 2050 under the RCP 4.5 and 8.5 emission scenarios. The study identified medium-maturity hybrids with extended grain-filling periods and high light use efficiency as promising traits for climate resilience, although the ideal trait combinations may vary across different environments. This modeling framework provides early indications of the timing, locations, and desired hybrid characteristics to policymakers and breeders, indicating the urgency for the development of climate-resilient maize hybrids [119]. Moreover, the general large area model (GLAM) has been evaluated for its capability to simulate regional climate and crop yields in Nigeria. GLAM uses daily climatic data from the Regional Climate Model (RegCM3) and indicates realistic simulations of the mean and spatial distribution of yields for maize, rice, cowpea, and groundnut, with generally <36% root mean square errors of observed yields. However, this model’s performance varies across ecological zones. Sensitivity analyses revealed simulated crop yields were sensitive to parameters like harvest index, extinction coefficient, optimum temperature, and transpiration efficiency, where extinction coefficient and transpiration efficiency had the most significant impact. Although RegCM3 can provide realistic climate simulations, with 0.72 to 0.96 correlations between observed and simulated variables, it can slightly underestimate rainfall and maximum temperature during the wet season. The study concluded that enhancing meteorological input data quality could further improve GLAM’s performance in regional crop yield simulations, highlighting its potential for assessing climate change impacts and informing adaptation strategies [214].

Many studies have been focused on integrating climate models with crop models. The CERES-Maize model was established to simulate the impact of climate change on maize yields in Heilongjiang Province, China. It identified significant yield reductions in some areas and suggested potential adaptation strategies, including adjusting planting dates and selecting appropriate cultivars [215]. A study employed phenology observations and the APSIM-Maize model to investigate the effect of changing sowing dates on maize production in northeast China (NEC) from 1981 to 2014. It was found that actual sowing dates were delayed by 1–6 days per decade, leading to shorter growing seasons and reduced solar radiation interception. Delaying sowing dates decreased potential maize yields by 0.6% per day across NEC. However, an increase in the sowing dates had mixed effects; for example, it enhanced potential yields in high latitudes (up to 1.6%) but reduced them in low latitudes (up to 2.7%) [216]. Furthermore, efforts have been made to integrate simplified crop models into existing decision support systems to enhance their accessibility and usability. For instance, the System Approach to Land Use Sustainability (SALUS) crop model was integrated into the Decision Support System for Agrotechnology Transfer (DSSAT) framework. This integration aimed to provide a less complex alternative for crop modeling while maintaining reliable predictions. Moreover, scientists have explored various parameter estimation techniques, including Bayesian approaches, to calibrate these models using both limited and detailed datasets. These studies have highlighted the importance of in-season data for accurate growth predictions and the potential of simplified models for simulating annual crop production when detailed information is scarce [217]. In addition, process-based models have long been used for evaluating the long-term effects of no-tillage (NT) and conventional tillage (CT) systems on GY and soil properties. A comprehensive study used the field data collected from 2006 to 2011 to calibrate and validate the CSM-CERES-Maize and CSM-CROPGRO-Soybean models. These models exhibited high accuracy in predicting crop yields under both tillage systems. Long-term simulations spanning 30 years revealed that NT systems enhanced soil organic carbon content, thereby improving crop yields and biomass production compared to CT systems. These findings highlight that NT applications offer sustainable benefits for soil health and crop productivity over extended periods. These data provide insights into the current sustainable agricultural practices and their long-term effects on soil quality and crop performance [218].

Crop modeling studies in sub-Saharan Africa (SSA) have also provided valuable insights into the potential impacts of climate change on agricultural productivity. Furthermore, a comprehensive multi-model assessment investigated the effects of varying temperatures, CO_2_ concentrations, and rainfall on maize yields in different nitrogen input levels and five SSA environments. The study employed 25 maize models, calibrated with the help of two-year experimental data from each country. The models demonstrated good accuracy in reproducing observed GY variations, with an average relative root mean square error of 26%. Furthermore, nitrogen fertilization was observed as a critical factor, which influenced the crop responses to climatic change. The research highlighted the importance of simulating daily soil nitrogen supply and leaching processes in low-input systems to accurately assess climate change impacts. These data indicate the complex interactions of climate change with nutrient management, thereby highlighting the need for robust adaptation strategies in SSA agriculture [219]. Therefore, these integrated modeling approaches can be utilized to assess the multifaceted impacts of climate change on maize production and develop an effective strategy to ensure the resilience of this vital crop.

### 1.10. Challenges and Future Directions

Navigating the complexities of climate change and its impacts on maize cultivation involves several technical challenges, which should be assessed while charting innovative future directions to ensure resilience and sustainability. One of the primary challenges is climate variability and the increasing frequency of extreme weather events. Temperature fluctuations, such as heat waves, can cause HS in maize, which can reduce crop yield and quality. Furthermore, variable rainfall patterns, such as prolonged droughts and intense storms, can also disrupt the maize growth cycle and result in crop failure. Additionally, extreme weather events such as hurricanes and floods can substantially damage maize fields, highlighting the need for more robust and resilient maize varieties. Moreover, soil degradation, which can be exacerbated by increased rainfall, can cause erosion and nutrient depletion from intensive farming practices, thereby further complicating maize cultivation by reducing soil fertility and sustainability. Pest and disease pressures are also intensifying with climate change as warmer temperatures and altered precipitation patterns create favorable conditions for pests like the fall armyworm, which significantly affects maize production. In addition, climate change can also shift plant disease prevalence and distribution, posing new threats to maize crops. Therefore, addressing these biotic stresses requires advanced integrated pest management strategies and disease-resistant maize varieties. It was also observed that limited genetic diversity in maize is another clinical challenge, which restricts the potential to develop varieties with enhanced resilience to climate stressors. The narrow genetic base of cultivated maize necessitates a significant investment in breeding programs and access to diverse genetic resources to develop climate-resilient varieties effectively.

Therefore, to overcome these challenges, climate-smart agricultural practices should be adopted. Conservation agriculture practices such as minimum tillage, crop rotation, and cover cropping have been observed to enhance soil health, improve water retention, and increase maize resilience to climate variability. These practices, along with integrated pest management strategies, can alleviate the effect of pests and diseases, as well as reduce the reliance on chemical pesticides. Advanced breeding techniques play a crucial role in developing climate-resilient maize varieties. GS accelerates the breeding process via more efficient identification and selection of desirable traits. CRISPR-Cas9 gene-editing technology, a powerful tool for developing resilient varieties, enables precise modifications to improve stress tolerance and pest resistance in maize. Enhancing genetic diversity through expanded germplasm collection and conservation is vital for broadening the genetic base available for breeding programs. International collaboration in maize breeding facilitates the exchange of genetic resources and knowledge, thereby promoting the development of resilient varieties. Furthermore, precision agriculture technologies are promising tools for optimizing maize cultivation. Moreover, maize fields can be more effectively monitored and managed by remote sensing and geographic information systems (GIS), thereby helping optimize resource use and improve yield predictions. Smart irrigation systems, using real-time data on soil moisture and weather conditions, enhance water use efficiency and reduce the risk of drought stress, ensuring sustainable water management in maize cultivation. Incorporating cutting-edge biotechnological approaches further strengthens maize resilience. In addition, gene identification via GWAS helps identify genetic markers linked with desirable traits. Furthermore, multiomics approaches, integrating transcriptomics, metabolomics, and proteomics, provide a comprehensive understanding of maize responses to environmental stresses at the molecular level. These data help the development of maize varieties with enhanced stress tolerance. Speed breeding and MAS expedite the breeding cycle, which allows rapid development and deployment of improved maize varieties. Therefore, strategies such as carbon sequestration mitigate climate by optimizing agricultural practices and reducing the overall impact of agriculture on climate change. Policy and institutional support are crucial for advancing climate-resilient maize research and the adoption of innovative practices. Increased funding for research will accelerate the development of novel approaches to address climate challenges. Moreover, strengthening agricultural extension services will ensure that farmers have access to the latest knowledge and technologies for climate-smart maize cultivation. Developing and implementing supportive policy frameworks incentivizes sustainable maize agriculture practices and promotes resilience. Therefore, these strategies should be integrated to enhance the resilience and sustainability of maize agriculture, as well as ensure food security and agricultural prosperity during climate change.

## 2. Conclusions

To effectively address challenges such as climate warming, shifts in precipitation patterns, and intensified pest and disease pressures, it is imperative to establish a systematic and coordinated approach spanning the entire continuum “from gene to field”.

The primary strategy involves the in-depth exploration and utilization of the rich genetic diversity present in wild relatives, landraces, and multi-parental populations, with a particular emphasis on the identification and pyramiding of key alleles conferring drought tolerance, heat resistance, and other vital agronomic traits. Furthermore, it is essential to precisely localize stably expressed QTLs, validated across multiple environments, down to their causal SNPs or genes. By integrating genomic selection and accelerated breeding technologies, the genetic improvement of complex stress-resistance traits can be expedited, thereby enhancing genetic gain. In addition, for confirmed bottleneck genes, targeted modification using gene editing tools such as CRISPR should be employed to achieve trait breakthroughs at the single-gene level. Meanwhile, incorporating high-throughput phenotyping data from drones, along with transcriptomic, metabolomic, and other multi-omics information, into predictive models can substantially improve selection accuracy. Finally, climate-smart agronomic practices, including deficit irrigation, mulching for soil moisture conservation, and precision fertilization, should be promoted in tandem with the deployment of improved cultivars. The integrated application of these complementary strategies will serve to effectively safeguard stable maize production under increasingly severe climatic fluctuations.

## Figures and Tables

**Table 1:** List of genes associated with different abiotic and biotic stress tolerance in maize.

Gene ID	Gene Description	References
*ZmVPP1*	Vascular pyrophosphatase improves drought tolerance	[18]
*ZmACA1*	The gene for cold stress tolerance	[19]
*ZmDREB2A*	The gene for cold stress tolerance	[20]
*ZmERF3*	The gene for cold stress tolerance	[19]
*ZmCOI6.1*	The gene for cold stress tolerance	[21]
*ZmPP2C2*	The gene for abiotic stress response	[22]
*ZmMKK4*	The gene for abiotic stress response	[23]
*GRMZM2G329229*	Associated with different stress conditions such as heat and drought	[24]
*GRMZM2G313009*	Associated with different stress conditions such as heat and drought	[24]
*GRMZM2G043764*	Associated with different stress conditions such as heat and drought	[24]
*GRMZM2G109651*	Associated with different stress conditions such as heat and drought	[24]
*GRMZM2G159307*	Encodes ATP binding protein; important for response to stresses	[25]
*GRMZM2G104325*	Encodes ATP binding protein; important for response to stresses	[25]
*Zm00001d048531*	Encodes an RNA helicase; associated with improved stress tolerance	[26]

**Table 2:** List of SNPs/QTLs/candidate genes identified by GWAS for multiple traits linked to physiology and yield.

Mapped Traits	Population Type	Sample Size	Number of SNPs/QTLs/Genes	Chromosomal Location	References
Corn earworm resistance	Diverse inbreed lines	287	51 SNPs	1, 2, 3, 4, 5, 6, 7, 8, 9, and 10	[69]
Gray leaf spot resistance	Diverse inbred lines	157	7 SNPs	1, 2, 3, 4, 5, 6, 7, and 10	[70]
Southern corn rust resistance	Diverse inbred lines	253	7 SNPs	4, 8, and 10	[71]
Corn ear rot resistance	Diverse inbred lines	242	5 candidate genes	5, 7, and 10	[72]
Ear rot resistance	Diverse inbred lines	244	8 candidate genes	1, 2, 3, 5, 7, and 9	[73]
Fumonisin accumulation in kernels	Diverse inbred lines	270	39 SNPs/17 QTLs	3 and 4	[74]
Maize lethal necrosis (MLN) and maize chlorotic mottle virus (MCMV)	Three double-haploid populations	965	54 SNPs and 40 QTLs	1, 2, 3, 4, 5, 6, 7, 8, and 9	[75]
Striga resistance	White maize inbred lines	132	24 SNPs	1, 3, 4, 5, 7, 8, 9, and 10	[76]
Maize leaf necrosis resistance	Diverse inbred lines	1400	32 SNPs and 9 candidate genes	1, 3, 4, 7, 9, and 10	[77]
Fusarium verticillioides resistance	Maize association population	230	42 SNPs and 25 candidate genes	1, 2, 3, 4, 5, 6, 7, 8, 9, and 10	[78]
Corn leaf blight	Association mapping panel	419	22 SNPs	1, 6, 7, 8, and 10	[79]
Aspergillus flavus resistance in kernels	Diverse inbred lines	313	4 SNPs and 16 candidate genes	1, 2, 8, and 9	[80]
Gray leaf spot resistance	Diverse inbred lines	410	22 SNPs	1, 2, 6, 7, and 8	[81]
Rough dwarf disease resistance	Diverse inbred lines	292	22 SNPs	1, 3, 4, 7, and 8	[82]
Striga resistance	Diverse inbred lines	141	22 SNPs	1, 3, 4, 5, 6, 7, 8, 9, and 10	[83]
Leaf streak resistance	Diverse inbred lines	200	11 SNPs	1, 2, 5, 7, 8 and 9	[84]
Root architecture traits	Diverse inbred lines	300	19 SNPs	1, 2, 5, 7, and 8	[69]
Leaf angle and leaf orientation	Diverse inbred lines	80	33 SNPs	1, 3, 4, 5, 6, 7, and 9	[85]
Leaf cuticular conductance	Diverse inbred lines	468	9 SNPs and 7 candidate genes	1, 4, 7, 8, and 10	[86]
Leaf angle	Diverse inbred lines	285	96 SNPs	1, 2, 3, 4, 5, 6, 7, 9, and 10	[87]
Chlorophyll content	Diverse inbred lines	378	19 SNPs	2, 4, 5, 6, and 10	[88]
Plant height	Maize hybrids	300	9 SNPs and 2 candidate genes	1, 2, 4, 7, 9, and 10	[89]
Tassel architecture	Association panel	359	55 candidate genes/19 QTLs	1, 2, 3, 4, 5, 6, 7, 8, 9, and 10	[90]
Kernal row number	Diverse inbred lines	639	49 candidate genes	1, 2, 3, 5, 9, and 10	[91]
Ear diameter	Multiple parent population	162	11 SNPs and 3 QTLs	1, 2, 3, 6, 8, and 9	[92]
Ear traits (ear length, diameter, kernel length and width, cob diameter)	Inbred association population	292	20 SNPs	1, 2, 3, 4, 5, 6, 7, 8, 9, and 10	[93]
Stalk strength	Diverse inbred lines	345	94 QTLs and 241 SNPs	1, 2, 3, 4, 5, 6, 7, 8, 9, and 10	[94]
Grain quality traits	Diverse inbred lines	248	49 SNPs and 29 candidate genes	1, 2, 3, 4, 5, 6, 7, 8, 9, and 10	[95]
Grain yield quality traits	Association mapping population	410	42 SNPs	1, 2, 3, 4, 5, 6, 7, 8, 9, and 10	[25]
Yield related traits	Diverse inbred lines	291	59 SNPs and 66 candidate genes	1, 2, 3, 4, 6, 7, 8, 9, and 10	[96]
Grain yield and other traits	Diverse inbred lines	169	40 SNPs and 6 candidate genes	1, 2, 8, and 10	[97]
Grain yield and flowering time	Inbred association panel	300	1549 SNPs and 46 candidate genes	1, 2, 4, 5, 8, and 10	[24]
Root architecture traits	RIL population	179	8 SNPs	1, 2, 4, and 10	[98]
Root hair length	Diverse inbred lines	281	11	1, 2, 4, 5, 6, and 10	[99]
Root hair length	Association panel	200	88 QTLs	1, 2, 3, 4, 5, 6, 7, 8, 9, and 10	[100]
Total root length	Diverse inbred lines	280	38 candidate genes	1, 2, 3, 4, 6, 7, 8, and 9	[101]
Drought tolerance	Diverse inbred lines	210	26 QTL promising candidate genes	1, 2, 5, 8, and 10	[102]
Drought tolerance	Association panel	379	15 candidate genes	1, 3, 4, 5, 6, 8, and 9	[103]
Drought and heat resistance	Diverse inbred lines	162	117 SNPs and 20 candidate genes	1, 2, 5, and 7	[26]
Heat tolerance	Double haploid lines	662	46 SNPs	1, 2, 3, 6, 7, and 8	[104]
Heat resistance	Diverse inbred lines	375	14 SNPs	1, 2, 4, 5, and 9	[105]
Thermos tolerance of seed	Diverse inbred lines	261	4 QTLs, 17 candidate genes, and 42 SNPs	1, 2, 3, 4, 5, 6, 7, 8, 9, and 10	[106]
Chilling tolerant	Diverse inbred lines	190	26 SNPs and 37 candidate genes	4, 6, 8, and 9	[107]
Cold tolerance	Diverse inbred lines	80	4 SNPs and 12 QTLs, 1 gene	3	[108]
Salt tolerance	Diverse inbred lines	150	7 SNPs and 8 candidate genes	1, 3, and 6	[109]
